# Comparison of different lymph node staging systems in patients with node-positive cervical squamous cell carcinoma following radical surgery

**DOI:** 10.7150/jca.48085

**Published:** 2020-10-23

**Authors:** Qinhao Guo, Jun Zhu, Yong Wu, Hao Wen, Lingfang Xia, Min Yu, Simin Wang, Xingzhu Ju, Xiaohua Wu

**Affiliations:** 1 Department of Oncology, Shanghai Medical College, Fudan University, 130 Dong-An Road, Shanghai 200032, China.; 2 Department of Gynecologic Oncology, Fudan University Shanghai Cancer Center, 270 Dong-An Road, Shanghai 200032, China.

**Keywords:** Akaike information criterion, CSCC, C index, LN staging systems, node-positive, prognostic value

## Abstract

**Objective:** We compared the prognostic accuracy of four lymph node (LN) staging systems - the 2018 International Federation of Gynecology and Obstetrics (FIGO) stage, number of positive lymph node (PLN), metastatic lymph node ratio (LNR), and log odds of positive lymph nodes (LODDS) systems - in patients with node-positive cervical squamous cell carcinoma (CSCC) following radical surgery and explored the optimal choice for clinical applications.

**Materials and methods:** Data were retrospectively collected from 928 node-positive CSCC patients who underwent radical surgery between 2006 and 2014 in our center. Tree-based recursive partitioning was applied to split variables (PLN, LNR, and LODDS) into low-risk and high-risk groups. The log-rank test was used to compare survival curves, and Cox regression analysis was performed to identify prognostic factors. The relative discriminative abilities of the different staging systems were assessed using Harrell's concordance index (C index) and the Akaike information criterion (AIC).

**Results:** The mean number of PLNs was 3.8 (range: 1-44 nodes). According to the 2018 FIGO staging system, 831 patients had stage IIIC1, and 97 had stage IIIC2. For the PLN system, 761 patients were included in the low-risk group, and 167 were included in the high-risk group. For the LNR system, 658 patients were included in the low-risk group, and 270 were included in the high-risk group. The low-risk LODDS group included 694 patients, while the high-risk LODDS group included 234 patients. All four staging systems had a significant influence on patients' progression-free survival (PFS, *P* < 0.001) and overall survival (OS, *P* < 0.001). Univariate analysis and multivariate Cox analysis adjusted for significant factors indicated that the four staging systems were significant prognostic factors for PFS and OS. Among them, the PLN system was noted to have the best prognostic performance for both PFS (C index: 0.582; AIC: 8213.33) and OS (C index: 0.624; AIC: 8433.80).

**Conclusion:** The PLN system seemed to be the most accurate LN staging method for predicting node-positive CSCC following radical surgery.

## Introduction

With the benefit of cervical cancer screening programs, the number of early cervical cancers detected has increased worldwide 1. For patients with International Federation of Gynecology and Obstetrics (FIGO) stage IB-IIA cervical cancer, radical hysterectomy with pelvic lymphadenectomy (RHPL) is considered the standard surgical treatment 2.

The presence of lymph node (LN) metastases has been shown to be an independent prognostic factor for progression-free survival (PFS) and overall survival (OS) in both early-stage disease and locally advanced-stage disease and has always been used to help guide treatment in terms of postoperative adjuvant treatment strategies 3. However, the FIGO staging system for cervical cancer did not consider LN status before the 2018 version. Based on previous studies on LN metastasis, the FIGO Committee added IIIC1 (pelvic LN metastasis only) and IIIC2 (para-aortic LN metastasis) to the new FIGO staging system for cervical cancer in 2018 4. Nevertheless, this current 2018 FIGO staging system for cervical cancer is based solely on the anatomic location of metastatic LNs and does not consider the number of LNs, which may limit the precision of its prognostic significance. LN status in many other solid tumors generally relies on a combination of the anatomic location and the number of involved nodes 5-7. Does the number of positive LNs affect the prognosis of patients with cervical cancer? In recent years, various studies have shown correlations between different LN staging systems and patient survival outcomes 8-11. Among them, the LN staging systems considering positive LNs include the number of positive lymph nodes (PLNs), the lymph node ratio (LNR), and the log odds of positive nodes (LODDS).

Do the LN staging systems including the number of LNs (PLN, LNR, and LODDS) have prognostic significance? Of the three different LN staging methods, the PLN, LNR, and LODDS systems, which is the most accurate for node-positive cervical squamous cell carcinoma (CSCC) patients? The prognostic value of the different LN staging systems in cervical cancer remains controversial, and to date, no studies have compared these systems in patients with CSCC, which is the most common pathological type (accounting for 80-90% of cases) of cervical cancer. Moreover, CSCC has a lower likelihood of LN metastasis than other pathological types 12. The purpose of this study was to evaluate and compare the prognostic value of the 2018 FIGO stage, PLN, LNR, and LODDS systems in node-positive CSCC patients initially treated with RHPL from a large cancer institution to identify the most accurate LN staging method.

## Methods

### Patients

A retrospective cohort study was conducted in the Department of Gynecologic Oncology, Fudan University Shanghai Cancer Center (China), which included patients with FIGO (2009) stage IB1-IIa2 CSCC who underwent radical abdominal hysterectomy with or without bilateral salpingo-oophorectomy and pelvic ± para-aortic lymphadenectomy from 2006 to 2014.

All the enrolled patients had undergone standard pelvic lymphadenectomy by experienced gynecological oncologists, which was reviewed from the electronic medical charts. All nodal and fatty tissues were removed between the external and internal iliac arteries from the bifurcation of the common iliac artery up to the circumflex vein and above the obturator nerve. If intraoperative palpation suggested suspicion of para-aortic LN involvement or if intraoperative frozen section examination showed positive para-aortic or standard iliac LNs, para-aortic LN resection was performed as previously described 13. All the microscopic slides were reviewed by the same gynecology-dedicated pathologist and were confirmed by a second experienced gynecologic pathologist. Patients who received neoadjuvant chemotherapy or preoperative radiotherapy and died within 30 days after surgery and those who had a follow-up time of less than three months were excluded from this study. All included patients received adjuvant radiotherapy or concurrent chemoradiotherapy. A monthly follow-up was ensured for the first 6 months after surgery. After treatment, patients were followed every three months for the first two years, every six months for the next three years, and once per year thereafter. Follow-up visits included pelvic examinations, abdominal ultrasonography, chest X-ray, routine blood tests, serum squamous cell carcinoma antigen (SCC-Ag) tests, vaginal cytology, and CT or MRI scans. Written informed consent was obtained from all the participants preoperatively. The study was approved by the Institutional Ethics Review Committee of Fudan University Shanghai Cancer Center, Shanghai, China.

### Classifications of LNs

The total number of removed LNs (RLNs), the anatomic locations of LNs, and the number of positive LNs were always documented in the medical records. For this study, four different classifications were used to further evaluate the prognostic role of LNs in CSCC patients. Tree-based recursive partitioning was applied to identify the optimal cutoff value to divide variables (PLN, LNR, and LODDS) into low-risk and high-risk groups. PLN was defined as the number of positive LNs, and it was divided into 2 groups: low-risk PLN (1 ≤ PLN ≤ 5) and high-risk PLN (PLN > 5). The LNR system was defined as the ratio between PLN and RLN, and subgroups were categorized as follows: low-risk LNR (0 < LNR ≤ 0.16) and high-risk LNR (0.16 < LNR ≤ 1). The LODDS system was defined determined as the log of the ratio between the number of PLNs and the number of negative nodes (NLNs), and its value was calculated by an empirical logistic formula: log ((PLN + 0.5)/(NLN+ 0.5)). Furthermore, 0.5 was added to both the numerator and denominator to avoid singularity. The LODDS system was classified as follows: low-risk LODDS (-1.54 < LODDS ≤ -0.61) and high-risk LODDS (-0.61 < LODDS ≤ 1.33). The 2018 FIGO staging system was defined according to the 2018 FIGO staging system for cervical cancer: 2018 FIGO stage IIIC1 referred to pelvic LN metastasis only, while 2018 FIGO stage IIIC2 represented para-aortic LN metastasis.

### Statistical analyses

Scatter plots with Pearson correlation coefficients were used to assess the correlation between the LODDS, LNR, and PLN systems. PFS was defined as the time of primary surgery to the first disease progression event, and OS was defined as the interval from the date of primary surgery to death or the latest observation. A log-rank test was performed to analyze correlations between patients' clinicopathologic characteristics and 5-year PFS and OS, and Kaplan-Meier survival curves were used to compare PFS and OS between different groups of patients. Hazard ratios (HRs) with 95% confidence intervals (CIs) were calculated by univariate and multivariate analyses using Cox proportional hazards models to evaluate prognostic factors for survival. First, univariable analysis was performed to identify which confounding factors, including age, menopausal status, FIGO stage (2009), tumor diameter, depth of stromal invasion, lymph-vascular space invasion (LVSI), parametrial invasion, vaginal margin invasion, 2018 FIGO stage, PLNs, LNRs, and LODDS, were prognostic factors. Second, to avoid collinearity and to reduce interference within the same multivariate model, we performed multivariate survival analysis adjusted for significant factors from the univariate analysis (*P* < 0.05) in different models with the inclusion of only one of these LN staging systems each time. The different models included the 2018 FIGO stage (Model 1), PLN (Model 2), LNR (Model 3), and LODDS (Model 4) systems separately, and Model 5 combined all of the LN staging systems. Two approaches to correctly evaluate and compare the relative discriminative abilities of the different LN staging methods were used: one based on estimation of Harrell's concordance index (C index) and another based on the Akaike information criterion (AIC). In general, a higher C index represents a better discrimination ability, and a predictive model with a lower AIC indicates a better model fit. *P* < 0.05 was considered to indicate statistical significance. All statistical analyses were performed with SPSS (version 22.0; SPSS Inc, Chicago, Ill) and R software (version 3.5.2).

## Results

### Clinical and pathological characteristics and survival analysis

A total of 928 patients with stage IB1-IIa2 CSCC were enrolled in the analysis. The mean age of these eligible patients was 46.58 years (range: 23-87). Among the patients, 129 (13.9%) and 49 (5.28%) had positive parametrial invasion and vaginal margin invasion, respectively (Table 1). The mean follow-up time was 35.7 months, with a range of 4-114 months. All the patients underwent radical abdominal surgery in our institution, 258 (27.80%) of whom underwent para-aortic lymphadenectomy. The mean number of RLNs was 23.55 (range: 1-70 nodes), and the mean number of PLNs was 3.8 (range: 1-44 nodes). According to the classification system described in the methods, when restaged by the 2018 FIGO staging system, 831 patients were in group IIIC1, and 97 were in group IIIC2. For the PLN system, 761 patients were in the low-risk group, and 167 were in the high-risk group. For the LNR system, 658 patients were in the low-risk group, and 270 were in the high-risk group. The low-risk LODDS group included 694 patients, while the high-risk LODDS group included 234 patients (Table 1).

**Figure 1:**
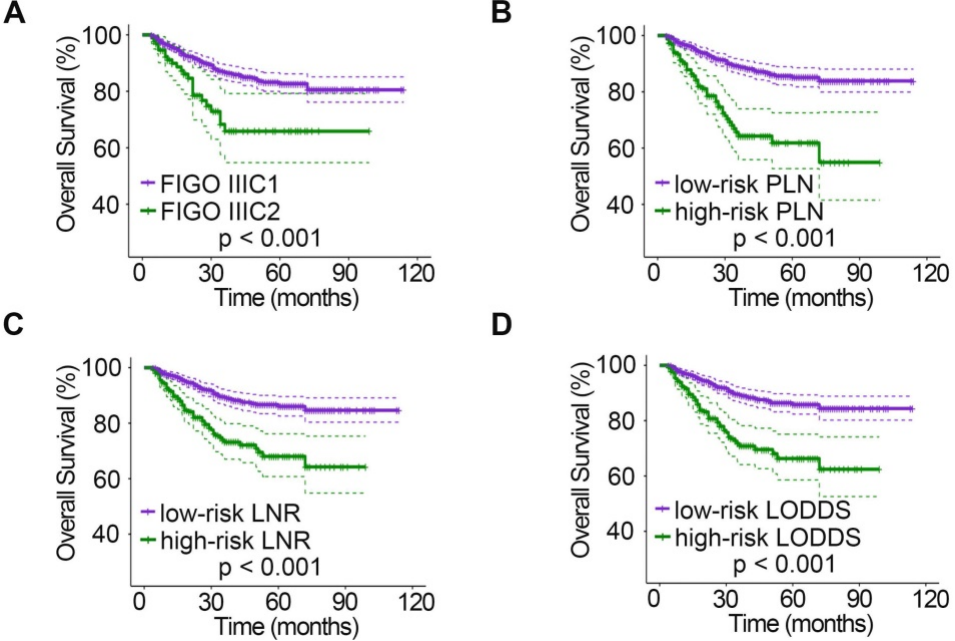
Kaplan-Meier curves for PFS stratified by LN categories based on 2018 FIGO stage (A), PLN (B), LNR (C), and LODDS (D). PFS, progression-free survival; LN, lymph node; FIGO, International Federation of Gynecology and Obstetrics; PLN, positive lymph node; LNR, lymph node ratio; LODDS, log odds of positive nodes.

**Figure 2:**
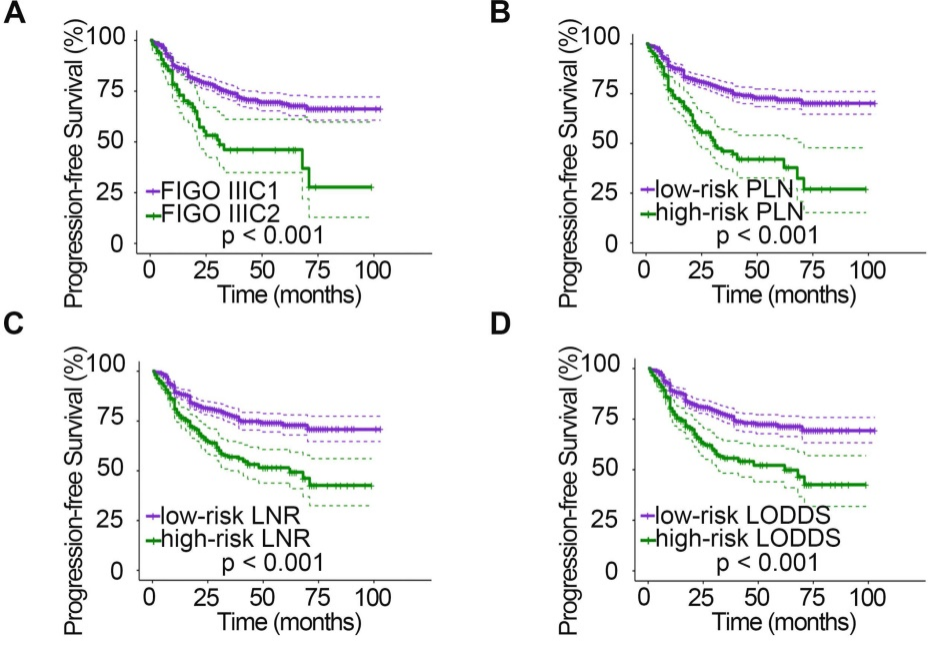
Kaplan-Meier curves for OS stratified by LN categories based on 2018 FIGO stage (A), PLN (B), LNR (C), and LODDS (D). OS, overall survival; LN, lymph node; FIGO, International Federation of Gynecology and Obstetrics; PLN, positive lymph node; LNR, lymph node ratio; LODDS, log odds of positive nodes.

The relationships between clinicopathologic characteristics and 5-year PFS and OS are shown in Table 1. Patients with stage IIa disease (P_PFS_ = 0.001, P_OS_ = 0.046), a tumor diameter > 4 cm (P_PFS_ = 0.006, P_OS_ = 0.004), deep stromal infiltration (P_PFS_ = 0.006, P_OS_ = 0.013), positive LVSI (P_PFS_ < 0.001, P_OS_ < 0.001), parametrial invasion (P_PFS_ < 0.001, P_OS_ < 0.001), and vaginal margin invasion (P_PFS_ < 0.001) tended to have a lower 5-year survival rate. In addition, when studying the relationship between different LN staging systems and patient survival, we found that the 2018 FIGO stage, PLN, LNR, and LODDS systems were all strongly associated with PFS and OS (all P < 0.001). However, patient age (P_PFS_ = 0.403, P_OS_ = 0.696) and menopausal status (P_PFS_ = 0.878, P_OS_ = 0.935) showed no significant association with survival. In addition, no significant association was found between vaginal margin invasion and patient OS (P = 0.357). The PFS (Fig. 1) and OS (Fig. 2) rates for the four LN staging systems were analyzed by Kaplan-Meier survival curves.

### Correlations between PLN, LNR, and LODDS

**Figure 3:**
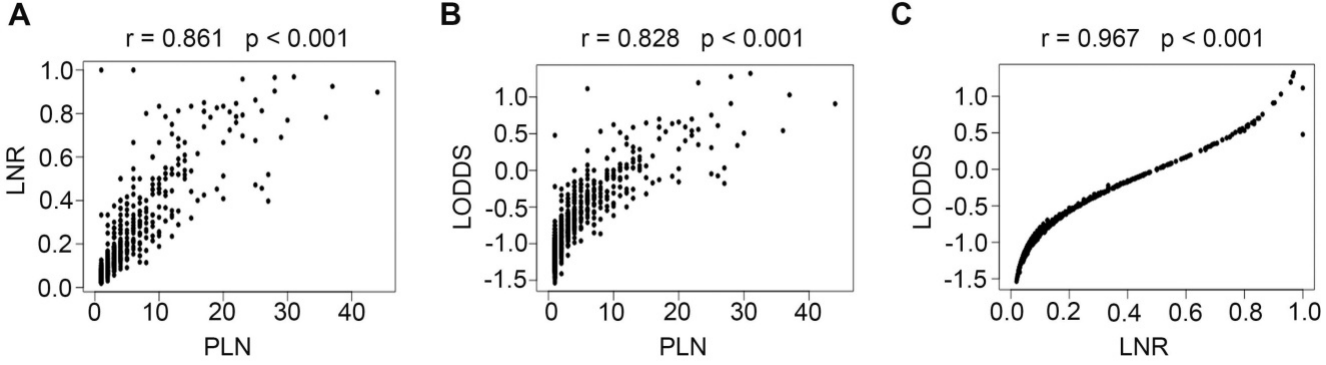
Correlations of PLN vs LNR (A), PLN vs LODDS (B), and LNR vs LODDS (C). PLN, positive lymph node; LNR, lymph node ratio; LODDS, log odds of positive nodes.

Scatter plots were created between any two variables of the PLN, LNR, and LODDS systems to evaluate the relationship between each of them. As shown in Fig. 3, a specific linear correlation was observed between any two variables (all *P* < 0.001), and the correlation coefficients were 0.861 (PLN and LNR), 0.828 (PLN and LODDS), and 0.967 (LNR and LODDS).

### Univariable and multivariable Cox models of prognostic factors for PFS and OS

Moreover, we used a Cox proportional hazards regression model to assess relationships between clinical and pathologic factors and PFS and OS. The univariable Cox model results showed that FIGO stage (2009) (P_PFS_ = 0.001, P_OS_ = 0.048), tumor diameter (P_PFS_ = 0.007, P_OS_ = 0.005), the depth of stromal invasion (P_PFS_ = 0.008, P_OS_ = 0.020), LVSI (P_PFS_ < 0.001, P_OS_ < 0.001), parametrial invasion (P_PFS_ < 0.001, P_OS_ < 0.001), 2018 FIGO stage (P_PFS_ < 0.001, P_OS_ < 0.001), PLNs (P_PFS_ < 0.001, P_OS_ < 0.001), LNRs (P_PFS_ < 0.001, P_OS_ < 0.001), and LODDS (P_PFS_ < 0.001, P_OS_ < 0.001) were all prognostic factors for PFS and OS. In addition to the above factors, the prognostic factors for PFS also included vaginal margin invasion (*P* = 0.001). However, age at diagnosis (P_PFS_ = 0.405, P_OS_ = 0.697) and menopausal status (P_PFS_ = 0.879, P_OS_ = 0.935) had no prognostic significance for PFS or OS, and vaginal margin invasion (*P* = 0.360) had no prognostic significance for OS (Table 2). The multivariable Cox model results showed that 2018 FIGO stage, PLNs, LNRs, and LODDS were all significant prognostic factors for PFS and OS in Model 1 (P_PFS_ = 0.001, P_OS_ = 0.031), Model 2 (P_PFS_ < 0.001, P_OS_ < 0.001), Model 3 (P_PFS_ < 0.001, P_OS_ < 0.001), and Model 4 (P_PFS_ < 0.001, P_OS_ < 0.001). In Model 5, however, when combining all the clinicopathologic parameters, only a PLN was shown to be an independent prognostic factor among the four LN staging systems for OS (*P* = 0.030, Table 3).

### Evaluation of the prognostic value of different LN staging systems

Through regression modeling, the LN staging system with the best prognostic discriminatory ability was then assessed using iterative statistical models and a comparison of C index and AIC values. When assessed using the established categorical cutoff values, the PLN system was noted to have the best prognostic performance for both PFS (C index: 0.582; AIC: 8213.33) and OS (C index: 0.624; AIC: 8433.80). To test whether the relative performance of the PLN, LNR, and LODDS systems was impacted by the chosen categorical cutoff values, repeat analyses were performed using continuous variables in the statistical models. When LN status was modeled as a continuous variable, for both PFS (C index: 0.617; AIC: 8209.37) and OS (C index: 0.657; AIC: 8433.60), the PLN system still outperformed the other nodal staging systems (Table 4).

## Discussion

For cervical cancer, the previous FIGO staging system before the 2018 FIGO staging system included only clinical and imaging parameters, thus representing only a “clinical staging system”. However, after considering the importance of LN status in the prognosis of patients, the new FIGO staging system included the status of LNs for the first time 4. The importance of the 2018 FIGO staging system has been confirmed by previous studies. A study by Yan et al. tested the prognostic ability of the new FIGO staging system. Their results showed that the 2018 FIGO staging system could help predict the survival of patients with high-risk factors after radical surgery 14. Consistent with their results, our study also revealed that the FIGO 2018 staging system was a significant prognostic factor for PFS and OS. However, the new FIGO staging system considers only the anatomic locations of positive LNs, resulting in some confusion in clinical practice. Whether patients with only pelvic LN metastasis but a more advanced stage (IIIc) might have a better prognosis than those with locally advanced diseases (IIB) remains controversial. According to the new FIGO staging system, cervical cancer patients with LN metastasis but without locally advanced tumors are all considered to have advanced diseases and might miss the opportunity for radical surgery. Therefore, appropriate and valuable LN staging systems must be explored to help guide the management of postoperative cervical cancer patients. The 2018 FIGO staging system lacks consideration of the number of positive LNs. In this study, the C index and AIC values indicated that the 2018 FIGO staging system was still inferior to the PLN, LNR, and LODDS systems, the other three staging systems associated with positive LNs (Table 4).

The PLN system is used as an LN evaluation method in the TNM staging system of many solid tumors 15, 16. Although PLNs are not included in the current staging system for cervical cancer, the TNM staging system for cervical cancer is only divided into N0 and N1 based on the presence or absence of LN metastasis. In recent years, the relationship between PLNs and the prognosis of cervical cancer patients has been clarified. In a study by Wang et al., patients treated with definitive concurrent chemoradiotherapy (CCRT) or intensity-modulated radiotherapy (IMRT) were included, and their results confirmed that the presence of PLNs (≥ 3) was an independent prognostic factor for OS, cancer-specific survival (CSS), and distant metastasis-free survival (DMFS) 17. Similarly, Kwon et al. also found that PLNs were associated with DMFS and disease-free survival (DFS) in patients with early-stage cervical cancer. The cutoff value for PLNs that they selected was three 18. In addition, Hosaka et al. compared the difference in OS between cervical cancer patients with one PLN and those with more than one PLN. They found that OS in patients with only one PLN was significantly superior to that of patients with multiple positive LNs 19. Consistent with the results described above, our study indicated that only the presence of PLNs was shown to be an independent prognostic factor among the four staging systems for PFS when all the clinicopathologic parameters were combined in a multivariate survival analysis (Table 3), and the PLN system was noted to have the best prognostic performance for both PFS and OS (Table 4).

In a retrospective study performed by Fleming et al., patients with stage I or II cervical cancer who had undergone radical hysterectomy and pelvic +/- para-aortic lymphadenectomy and were identified as LN positive after surgery were included, and the authors concluded that an LNR > 6.6% was associated with worse PFS, while an LNR > 7.6% was correlated with worse OS 8. In our study, the LODDS and LNR systems showed a high degree of consistency in node-positive patients (r = 0.967, Fig. 3), but many studies have reported that the LODDS system is superior to the LNR system in non-small cell lung cancer 20, breast cancer 21, oral squamous cell carcinoma 22, and gastric cancer patients 23. For cervical cancer, one study compared the prognostic value of the PLN, LNR, and LODDS systems in 50 high-risk cervical cancer patients treated with radical surgery and adjuvant treatment. In that study, the LODDS was the only significant prognostic factor for both DFS and OS 9. However, the LODDS was also a strong predictive factor in our study but did not show any superiority over the LNR. Similar results have also been reported in pancreatic head cancer 24.

The PLN system rather than the RLN system was shown to be the best nodal staging system in our research, leading to the controversial question: is the prognosis better with more RLNs? Previous studies have reached different conclusions on this issue. Shah et al. found that node-negative patients who underwent more extensive lymphadenectomy had better survival after including 5522 patients with stage IA2-IIA cervical cancer who underwent RHPL 25. In another retrospective study, Kim et al. found that an increased number of RLNs was associated with better survival in patients treated with surgery 26. Pieterse et al. found improved survival in LN-positive patients with a higher number of RLNs but noted no relationship between the number of RLNs and survival for LN-negative patients 27. However, in another two studies in which patients were also separated into LN-positive and LN-negative groups, more extensive lymphadenectomy had no positive effect on survival among node-positive patients and node-negative patients 28, 29. The inconsistent conclusions reported by these studies may be related to the use of different inclusion criteria, such as patients' physical condition, comorbidities, pathological type, and tumor stage. Additionally, the surgical skills of surgeons are important.

Different studies have selected different cutoff values when using the PLN system as the staging method 17-19. In this study, we used the method of tree-based recursive partitioning, which can help select the parameters that provide the optimal split for censored data 30. To further confirm this result, we performed the same analysis including the presence of PLNs, the LNR, and the LODDS as continuous variables, and the results still showed that the PLN system was the best staging method. Our results indicate that positive LNs have a significant impact on the prognosis of patients and should be removed as thoroughly as possible. However, excessive removal of negative LNs does not have a significant impact on the prognosis of patients but increases the risk of complications after surgery 31. Is thorough lymphadenectomy required for all cervical cancer patients, and can “low-risk” patients who can undergo small-scale lymphadenectomy or even avoid lymphadenectomy be screened out? At present, no specific methods are available to confirm LN metastasis before surgery. The accuracy of CT, MRI, and PET-CT is still not satisfactory 32. Fortunately, sentinel lymph node (SLN) biopsy, a method of intraoperative assessment, is increasingly being used in the standard management of early-stage cervical cancer. A series of studies have confirmed the high sensitivity and negative predictive value of SLN biopsy 33, 34. As a result, the National Comprehensive Cancer Network (NCCN) guidelines also recommend performing SLN biopsy as an alternative option for LN staging in the early stages of cervical cancer 35. However, the safety and accuracy of SLN biopsy may need to be verified by more large-scale and prospective studies. At the same time, SLN biopsy has higher requirements for pathology, which may also affect its application. In addition, several lines of evidence support the possibility of using specific biomarkers to improve early diagnosis and to evaluate the local and peripheral spread behavior, progression tendency and aggressiveness of cervical cancer, allowing surgeons to determine the best multidisciplinary approach and thus offer a better prognosis to patients 36. Based on growing evidence, biomarkers may significantly enhance the possibility of tailored management of cervical cancer. We believe that novel biomarkers can be found in the near future to accurately predict LN metastasis and thus achieve accurate navigation of LN resection. In addition to biomarkers, we may also find some new favorable evidence from routine programs, such as cervical glandular cytology and cervical conization 37, 38.

The development of medical science has changed rapidly, and we must continually review these data. Whether patients can obtain the best benefit from existing treatments is our eternal goal. With the gradual development of precision medicine, the management of cervical cancer should be personalized considering the performance status of patients, particularly elderly women. Some studies have shown that elderly patients can benefit from standard treatments in managing their gynecological cancers and should be treated in the same manner as younger patients 39, 40. However, a patient's physical condition is a factor that cannot be ignored, and the extent of surgery may always be tailored to the patient's performance status. If we can accurately stratify patients, elderly patients with poor health can safely avoid extensive lymphadenectomy without affecting their prognosis, representing the best of both worlds.

Several limitations existed in this study. First, both the clinical and pathological data were obtained from a single institution, which does not account for the diversity of treatments at other centers. In addition, our study included only IB1-IIa2 CSCC patients, although the incidence of squamous cell carcinoma is relatively high among all pathological types of cervical cancer. Notably, in contrast to CSCC, cervical cancer of other pathological types was not included in this study. More studies are needed to determine the relationship between the number of RLNs and patient survival in these pathological types.

## Conclusions

The 2018 FIGO stage, PLN, LNR, and LODDS systems appear to be independent prognostic factors. However, the PLN system seemed to be the most accurate and valuable LN staging method in patients with CSCC receiving radical surgery. The existing FIGO staging system has achieved significant progress compared with the previous version, but our results showed that the PLN system was superior to the 2018 FIGO staging system in terms of LNs alone. More multicenter and extensive sample studies are necessary to explore the combination of the PLN and FIGO staging systems.

## Figures and Tables

**Table 1:** Clinical and pathological characteristics and survival analysis of the patients (n=928)

	N	5-y PFS%	*p*-value	5-y OS%	*p*-value
**Age, Years**			0.403		0.696
≤50	629 (67.8%)	71.400		82.200	
>50	299 (32.2%)	66.300		78.400	
**Menopausal Status**			0.878		0.935
Premenopausal	656 (70.7%)	70.800		81.500	
Postmenopausal	272 (29.3%)	67.100		79.600	
**FIGO Stage (2009)**			0.001		0.046
IB	364 (39.2%)	76.900		84.500	
IIA	564 (60.8%)	65.000		78.800	
Tumor Diameter (cm)^*^			0.006		0.004
≤4	544 (59.7%)	71.700		84.300	
>4	367 (40.3%)	67.300		75.400	
Depth of Stromal Invasion^*^			0.006		0.013
<1/2	85 (9.2%)	87.100		94.300	
≥1/2	838 (90.8%)	67.800		79.500	
LVSI^*^			<0.001		<0.001
Negative	206 (22.9%)	82.1		92.7	
Positive	692 (77.1%)	65.100		77.300	
Parametrial Invasion*			<0.001		<0.001
Negative	784 (85.9%)	73.600		83.100	
Positive	129 (14.1%)	46.400		62.200	
Vaginal Margin Invasion^*^			0.001		0.357
Negative	861 (94.6%)	70.500		81.100	
Positive	49 (5.4%)	48.600		78.400	
**2018 FIGO Stage**			<0.001		<0.001
IIIc1	831 (89.5%)	71.900		82.700	
IIIc2	97 (10.5%)	50.500		65.800	
**PLN**			<0.001		<0.001
Low-risk (1≤PLN≤5)	761 (82.0%)	74.300		85.100	
High-risk (PLN>5)	167 (18.0%)	48.400		61.800	
**LNR**			<0.001		<0.001
Low-risk (0<LNR≤0.16)	658 (70.9%)	75.000		86.000	
High-risk (0.16<LNR≤1)	270 (29.1%)	56.700		68.000	
**LODDS**			<0.001		<0.001
Low-risk (-1.54<LODDS≤-0.61)	694 (74.8%)	74.100		85.800	
High-risk (-0.61<LODDS≤1.33)	234 (25.2%)	56.600		66.300	

PFS, progression-free survival; OS, overall survival; FIGO, International Federation of Gynecology and Obstetrics; LVSI, lymph-vascular space invasion; PLN, positive lymph node; LNR, lymph node ratio; LODDS, log odds of positive nodes. *Some parameters were not available in selected cases (see the numbers).

**Table 2:** Univariable Cox model of prognostic factors for PFS and OS

	Univariate (PFS)			Univariate (OS)		
	HR	95% CI	*p*-value	HR	95% CI	*p*-value
Age, Years	1.126	0.852-1.489	0.405	1.077	0.742-1.563	0.697
Menopausal Status	1.023	0.765-1.368	0.879	0.984	0.667-1.451	0.935
FIGO Stage (2009)	1.636	1.223-2.189	0.001	1.464	1.003-2.136	0.048
Tumor Diameter (cm)	1.454	1.109-1.905	0.007	1.665	1.167-2.375	0.005
Depth of Stromal Invasion	2.374	1.258-4.479	0.008	3.266	1.206-8.847	0.02
LVSI	2.033	1.381-2.991	<0.001	3.114	1.675-5.792	<0.001
Parametrial Invasion	2.48	1.820-3.378	<0.001	2.644	1.779-3.929	<0.001
Vaginal Margin Invasion	2.142	1.352-3.396	0.001	1.398	0.682-2.864	0.36
2018 FIGO Stage	2.494	1.768-3.518	<0.001	2.364	1.501-3.723	<0.001
PLN	2.836	2.133-3.771	<0.001	3.275	2.270-4.725	<0.001
LNR	2.335	1.784-3.055	<0.001	2.693	1.890-3.837	<0.001
LODDS	2.257	1.716-2.968	<0.001	2.851	1.998-4.068	<0.001

PFS, progression-free survival; OS, overall survival; FIGO, International Federation of Gynecology and Obstetrics; LVSI, lymph-vascular space invasion; PLN, positive lymph node; LNR, lymph node ratio; LODDS, log odds of positive nodes.

**Table 3:** Multivariable Cox model of prognostic factors for PFS and OS

	Multivariable (PFS)^a^			Multivariable (OS)^b^		
	HR	95% CI	*p*-value	HR	95% CI	*p*-value
**2018 FIGO Stage (Model 1)**	1.836	1.266-2.662	0.001	1.704	1.051-2.760	0.031
**PLN (Model 2)**	2.085	1.527-2.846	<0.001	2.487	1.677-3.687	<0.001
**LNR (Model 3)**	1.805	1.355-2.404	<0.001	2.131	1.467-3.095	<0.001
**LODDS (Model 4)**	1.705	1.273-2.284	<0.001	2.269	1.559-3.302	<0.001
**Different LN staging systems (Model 5)**						
2018 FIGO Stage	1.259	0.819-1.933	0.294	1.051	0.609-1.812	0.858
PLN	1.829	1.060-3.158	0.03	1.754	0.916-3.356	0.09
LNR	1.779	0.947-3.343	0.073	1.029	0.368-2.876	0.956
LODDS	1.663	0.809-3.418	0.167	1.515	0.515-4.463	0.451

PFS, progression-free survival; OS, overall survival; FIGO, International Federation of Gynecology and Obstetrics; LVSI, lymph-vascular space invasion; PLN, positive lymph node; LNR, lymph node ratio; LODDS, log odds of positive nodes; LN, lymph node. a: PFS adjusted for FIGO Stage (2009), Tumor Diameter (cm), Depth of Stromal Invasion, LVSI, Parametrial Invasion, and Vaginal Margin Invasion. b: OS adjusted for FIGO Stage (2009), Tumor Diameter (cm), Depth of Stromal Invasion, LVSI, and Parametrial Invasion.

**Table 4:** Evaluation of the prognostic value of different LN staging systems

	PFS		OS	
	C index	AIC	C index	AIC
2018 FIGO Stage	0.544	8218.38	0.553	8442.53
PLN (categorical)	0.582	8213.33	0.624	8433.80
LNR (categorical)	0.578	8220.21	0.62	8439.21
LODDS (categorical)	0.576	8219.98	0.62	8440.16
PLN (continuous)	0.617	8209.37	0.657	8433.60
LNR (continuous)	0.613	8218.06	0.651	8435.61
LODDS (continuous)	0.612	8218.14	0.653	8437.70

PFS, progression-free survival; OS, overall survival; LN, lymph node; FIGO, International Federation of Gynecology and Obstetrics; PLN, positive lymph node; LNR, lymph node ratio; LODDS, log odds of positive nodes; C index, Harrell's concordance index; AIC, Akaike information criterion.

## Abbreviations

FIGO: International Federation of Gynecology and Obstetrics
RHPL: radical hysterectomy with pelvic lymphadenectomy
LN: lymph node
PFS: progression-free survival
OS: overall survival
PLN: positive lymph node
LNR: lymph node ratio
LODDS: log odds of positive nodes
CSCC: cervical squamous cell carcinoma
SCC-Ag: serum squamous cell carcinoma antigen
RLN: removed lymph nodes
HR: hazard ratio
CI: confidence interval
LVSI: lymph-vascular space invasion
C index: Harrell's concordance index
AIC: Akaike information criterion
CCRT: concurrent chemoradiotherapy
IMRT: intensity-modulated radiotherapy
CSS: cancer-specific survival
DMFS: distant metastasis-free survival
DFS: disease-free survival
